# Regulation of breast cancer metastasis by Runx2 and estrogen signaling: the role of SNAI2

**DOI:** 10.1186/bcr3073

**Published:** 2011-12-09

**Authors:** Nyam-Osor Chimge, Sanjeev K Baniwal, Gillian H Little, Yi-bu Chen, Michael Kahn, Debu Tripathy, Zea Borok, Baruch Frenkel

**Affiliations:** 1 Department of Biochemistry & Molecular Biology, Keck School of Medicine of the University of Southern California, 2250 Alcazar Street, Los Angeles, CA 90033 USA; 2 Institute for Genetic Medicine, Keck School of Medicine of the University of Southern California, 2250 Alcazar Street, Los Angeles, CA 90033 USA; 3 Department of Orthopedic Surgery, Keck School of Medicine of the University of Southern California, 1200 N. State Street, Los Angeles, CA 90033 USA; 4 Bioinformatics Service Program, Norris Medical Library, University of Southern California, 2003 Zonal Ave, Los Angeles, CA 90089 USA; 5 Department of Medicine, Keck School of Medicine of the University of Southern California, 1441 East Lake Ave, Los Angeles, CA 90033 USA; 6 Will Rogers Institute Pulmonary Research Center, Keck School of Medicine of the University of Southern California, 2020 Zonal Avenue, Los Angeles, CA 90033 USA

## Abstract

**Introduction:**

In contrast to its role in breast cancer (BCa) initiation, estrogen signaling has a protective effect in later stages, where estrogen receptor (ER)α loss associates with aggressive metastatic disease. We asked whether the beneficial effect of estrogen signaling in late-stage BCa is attributable to the recently reported estrogen-mediated antagonism of the pro-metastatic transcription factor Runx2.

**Methods:**

MCF7/Rx2^dox ^breast cancer cells were engineered with a lentivirus expressing Runx2 in response to doxycycline (dox). Cells treated with dox and/or estradiol (E2) were subjected to genome-wide expression profiling, RT-qPCR analysis of specific genes, and Matrigel™ invasion assays. Knockdown of genes of interest was performed using lentiviruses expressing appropriate shRNAs, either constitutively or in response to dox. Gene expression in BCa tumors was investigated using a cohort of 557 patients compiled from publicly available datasets. Association of gene expression with clinical metastasis was assessed by dichotomizing patients into those expressing genes of interest at either high or low levels, and comparing the respective Kaplan-Meier curves of metastasis-free survival.

**Results:**

Runx2 induced epithelial-mesenchymal transition (EMT) evidenced by acquisition of a fibroblastic morphology, decreased expression of E-cadherin, increased expression of vimentin and invasiveness. Runx2 stimulated SNAI2 expression in a WNT- and transforming growth factor (TGF)β-dependent manner, and knockdown of SNAI2 abrogated the pro-metastatic activities of Runx2. E2 antagonized the pro-metastatic activities of Runx2, including SNAI2 upregulation. In primary BCa tumors, Runx2 activity, SNAI2 expression, and metastasis were positively correlated, and SNAI2 expression was negatively correlated with ERα. However, the negative correlation between SNAI2 and ERα in bone-seeking BCa cells was weaker than the respective negative correlation in tumors seeking lung. Furthermore, the absence of ERα in primary tumors was associated with lung- and brain- but not with bone metastasis, and tumor biopsies from bone metastatic sites displayed the unusual combination of high Runx2/SNAI2 and high ERα expression.

**Conclusions:**

E2 antagonizes Runx2-induced EMT and invasiveness of BCa cells, partly through attenuating expression of SNAI2, a Runx2 target required for mediating its pro-metastatic property. That ERα loss promotes non-osseous metastasis by unleashing Runx2/SNAI2 is supported by the negative correlation observed in corresponding tumors. Unknown mechanisms in bone-seeking BCa allow high Runx2/SNAI2 expression despite high ERα level

## Introduction

Metastasis of primary tumors to distant sites is a complex process that involves a sequence of interdependent events including intravasation, survival within the circulation, extravasation, and colonization. Epithelial-mesenchymal transition (EMT) has been strongly implicated in metastasis [1,2]. During EMT, epithelial cells dissociate from each other, in part due to loss of E-cadherin expression, upregulate mesenchymal markers, acquire a fibroblast-like morphology, reorganize their cytoskeleton, and become more motile and invasive [1,2]. Several transcription factors, including members of the SNAI family have been shown to promote EMT and thus tumor dissemination [3-5]. Bone metastasis is a frequent complication of breast cancer (BCa), with distinct gene signatures defining bone-seeking tumors [6-9].

Prolonged exposure to estradiol (E2) is associated with an increased risk of BCa [10-14]. The mechanisms through which estrogens contribute to BCa initiation and progression are complex and implicate estrogen receptor (ER)-mediated genomic and nongenomic signaling as well as the action of genotoxic estrogen metabolites [12]. In contrast to E2-mediated carcinogenesis, the presence of ERα is a favorable prognostic marker associated with less invasive tumors, and those negative for ERα are more aggressive [15]. A randomized clinical trial of postmenopausal women receiving equine estrogen treatment revealed a decrease in BCa incidence [16] and low-dose estradiol treatment has been proposed as a treatment modality for advanced ERα-positive BCa that does not respond to aromatase inhibition [17]. Indeed, introduction of ERα into ERα-negative BCa cells attenuated their pro-cancerous properties *in vitro *[18-21]. Thus, anti-estrogen therapy for BCa patients, while antagonizing the oncogenic properties of estrogens, may inadvertently result in loss of their anti-metastatic property. Better understanding of mechanisms underlying the beneficial role of estrogen signaling in advanced disease may inform the development of novel therapeutic approaches and improved treatment plans for BCa patients.

Runx2 is a lineage-specific transcription factor with crucial roles in both bone biology and carcinogenesis [22-24]. During development Runx2 is involved in the process of osteogenesis. Targeted disruption of Runx2 in mice leads to failure of osteoblast differentiation and bone formation [25,26], and Runx2 haploinsufficiency in humans results in the skeletal disorder cleidocranial dysplasia, with a similar phenotype observed in Runx2 haploinsufficient mice [25]. Although Runx proteins have tumor suppressor properties [24], recent studies assigned a role for Runx2 in promoting breast and prostate cancer metastasis [27-32]. Thus, Runx2 and E2 signaling play dual roles in BCa, with each functioning to either promote or suppress tumor progression. The mechanisms underlying these contrasting manifestations in cancer are poorly understood.

We previously showed that in the presence of ligand, ERα physically binds Runx2 and inhibits expression of several Runx2 target genes [33]; our recent study revealed that in MCF7 BCa cells E2 independently regulated about half of the Runx2-responsive genes [34]. We therefore hypothesized that gene(s) stimulated by Runx2 and inhibited by E2 may contribute to manifestations of the pro-metastatic or tumor suppressor functions of Runx2 and E2, respectively. Using a combination of tissue culture modeling and bioinformatics analysis of gene expression in BCa biopsies, we present evidence suggesting that SNAI2/SLUG plays a role in mediating the pro- and anti-metastatic effects of Runx2 and E2, respectively.

## Materials and methods

### Cell culture assays and reagents

MCF7 and T47D BCa cells were obtained from the American Type Culture Collection (Rockville, MD, USA). MCF7/Rx2^dox ^cells, engineered to express Runx2 in response to doxycycline (dox) [34], were maintained in Dulbecco's modified Eagle's medium (DMEM) containing 10% fetal bovine serum (FBS) and 50 μg/ml Hygromycin B (GIBCO, Carlsbad, CA). Data presented herein were obtained from cells cultured in phenol red-free DMEM containing 10% charcoal-stripped FBS (Hyclone, South Logan, UT), 0.5 μg/ml dox (Calbiochem, San Diego, CA) and/or 10 nM E2 (Sigma, St Louis, MO). For invasion assays, cells further engineered to constitutively express luciferase were placed in Matrigel™-containing inserts (BD Biosciences, Bedford, MA) and invasion was assessed based on luciferase activity in cells that had crossed the membrane [30]. The Wnt inhibitor ICG-001 was synthesized as previously described [35]. Mouse monoclonal anti-SNAI2 and anti-Runx2 antibodies were from Millipore (Billerica, MA) and Invitrogen (Grand Island, NY), respectively. Anti-TGF-β type I receptor (TGFBRI) blocking antibody (sc-398) and negative control antibody (sc-2027) were from Santa Cruz Biotechnology Inc. (Santa Cruz, CA).

### Real-time quantitative RT-qPCR analysis

Total RNA was extracted using Aurum™ Total RNA Mini-Kit (BioRad, Hercules, CA) and cDNA was synthesized with iScript Reverse Transcription Kit (BioRad). RT-qPCR was carried out in triplicate using iQ™ SYBR Green Supermix (BioRad) and a CFX96 qPCR machine (BioRad). All transcript levels were normalized to that of GAPDH. Primers used for PCR are listed in Additional file 1.

### Gene silencing

T47D/shRx2^dox ^cells, conditionally expressing shRNA to silence Runx2 in response to dox, were previously described [36]. For SNAI2 silencing, specific Mission-shRNA lentiviral plasmids (SHCLNG) and a negative control shRNA plasmid (SHC002) were purchased from Sigma and packaged as described [30] using HEK293T cells and the helper plasmids pMD.G1 and pCMV.R8.91. Viral particles were used for transduction of MCF7/Rx2^dox ^cells followed by selection with 1 μg/ml Puromycin (Invitrogen, Carlsbad, CA). Of five shRNAs tested, two were found to reduce SNAI2 expression by > 75% and the corresponding cells were propagated as described above. shRNA sequences used in this study are listed in Additional file 1.

### Data mining

For primary analysis of gene expression profiles in clinical samples, we compiled microarray data from 557 BCa patients by combining three Gene Expression Omnibus (GEO) datasets, GSE2034, GSE2603 and GSE12276 [37-39]. These datasets were generated using Affymetrix chips HG-U133A (GSE2034 and GSE2603) and HG-U133plus2 (GSE12276). The Affymetrix CEL files were downloaded into Partek, normalized using the RMA (Robust Multiarray Averaging) method and filtered for common probes using interplatform comparison. The data were standardized by centering and scaling and patients were dichotomized into low and high expressing groups with regard to the indicated genes of interest. Dichotomizing of the tumors according to the ESR1 Affymetrix probe '205225_at' was consistent with the histological definition of their ER status in 329 of 368 cases (89.4%) for which pathological annotation was available, and with the molecular definition of their ER status in 177 of 189 cases (93.7%) where it was based on different processing of the microarray data [9]. Clinical features, including hormonal status, metastasis-free survival time and sites of metastasis, have been compiled from the supplementary information of the published studies [9,37]. For each patient, metastasis-free survival was defined as the time interval between surgery and the diagnosis of metastasis. Only tumors with unambiguous information about sites of metastasis were included in the cohort. For metastatic tissue analyses we used the microarray data from 58 archival human breast carcinoma metastasis specimens deposited into GEO under accession number GSE14020 [9]. Because all the data were from publicly available resources, we did not require ethical approval to carry out this study.

Survival analyses and hierarchical clustering were performed using Partek Genomics Suite 6.6 and the correlation study was performed using Graphpad. Functional annotation of gene groups was performed using both the web-based Database for Annotation, Visualization, and Integrated Discovery (DAVID) system [40] and the Ingenuity Pathways Analysis (IPA™) package.

## Results

### Metastasis-related genes in BCa cells are stimulated by Runx2 and inhibited by E2

A recent study with prostate cancer cells and a dox-inducible lentiviral system identified a Runx2-regulated metastasis-related gene network [30]. We used the same system to conditionally express Runx2 in the ER-positive/Runx2-negative MCF7 BCa cells, and determined the genes that were responsive to Runx2 (*N *= 1,081) or E2 (*N *= 2,435) [34]. Interestingly, almost half of the Runx2 responsive genes were also E2-responsive (*N *= 530) and these 'common' genes are listed in Additional file 2 along with their level of responsiveness to Runx2 and E2. Functional computational analysis linked these 530 'common' genes to angiogenesis and cell movement (Table 1), suggesting their importance in tumor metastasis. A literature survey for the top ten 'common' genes (Table 2) identified three -- SNAI2, S100A9 and CXCL12, which have been implicated in cell motility and BCa metastasis [4,41,42]. Moreover, SNAI2 and S100A9 were downregulated by E2. These genes may contribute to the pro- and anti-invasive properties of Runx2 and E2, respectively.

**Table 1:** Functional Annotation Clustering of Genes Independently Regulated by Runx2 and E2 in MCF7/Rx2^dox ^Cells

Category	Term	*P *value
*Annotation Cluster 1*	*Enrichment Score: 4.50*	
GOTERM_BP_FAT	GO:0001944~vasculature development	2.22E-05
GOTERM_BP_FAT	GO:0048514~blood vessel morphogenesis	2.72E-05
GOTERM_BP_FAT	GO:0001568~blood vessel development	5.40E-05
		
*Annotation Cluster 2*	*Enrichment Score: 4.48*	
GOTERM_BP_FAT	GO:0016477~cell migration	2.55E-05
GOTERM_BP_FAT	GO:0048870~cell motility	3.77E-05
GOTERM_BP_FAT	GO:0051674~localization of cell	3.77E-05

DAVID Functional Clustering brings the similar, redundant, and heterogeneous annotation contents from the same or different resources into annotation groups. Enrichment Score ranks the significance of biological functions and measured as geometric mean of member's *P*-values in a corresponding annotation cluster. Shown are the top two annotation clusters for the 530 'common' genes, those independently regulated by Runx2 and E2.

**Table 2:** Genes Independently Regulated by Runx2 and E2 in MCF7/Rx2^dox ^Cells

Genes	dox (Runx2)/Control		E2/Control	
	
	fold change	*p *value	fold change	*P *value
RIS1	6.81	5.94E-08	1.53	7.19E-04
SNAI2	6.59	1.87E-07	-3.38	4.82E-06
SGK	4.77	1.91E-09	4.81	3.60E-09
C20orf114	4.77	1.87E-07	-1.52	6.72E-04
C10orf81	4.61	2.20E-07	-2.16	2.43E-05
MGP	4.52	1.37E-07	5.59	1.21E-07
CRISPLD2	4.49	7.78E-08	-1.96	1.72E-05
S100A9	3.98	4.19E-06	-1.59	3.28E-03
PLAC8	3.83	9.95E-07	-1.60	7.39E-04
CXCL12	3.67	1.99E-07	3.58	4.32E-07

Of 530 'Common Genes' (Supplemental Table 2), the ten most strongly stimulated by Runx2 are listed along with fold expression change in response to Runx2 and E2, as well as the corresponding *p *values. Negative values indicate repression.

### Functional link between Runx2, E2 and SNAI2 in BCa

**Figure 1:**
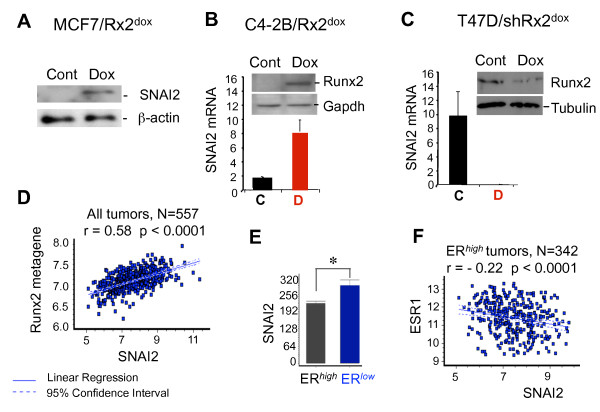
**Relationships between Runx2, E2 and SNAI2 in BCa**. **(A) **Runx2 was induced by treating MCF7/Rx2^dox ^BCa cells with dox for 48 hours, and the effect on SNAI2 protein level was assessed by Western blot analysis of nuclear extracts. (**B-C) **Runx2 was either induced for 48 hours by dox treatment of C4-2B/Rx2^dox ^PCa cells **(B) **or silenced by 48-hour dox treatment of T47D/shRx2^dox ^BCa cells **(C)**, and the effects on SNAI2 expression were analyzed by RT-qPCR. Western blot analysis of whole cell extracts confirms the respective dox-induced induction and silencing of Runx2. (**D) **Correlation between SNAI2 mRNA and Runx2 activity in a cohort of 557 BCa tumors. Runx2 activity was defined as the average normalized expression of genes that Runx2 stimulated by ≥ 2 fold in the MCF7/Rx2^dox ^cell culture model, except SNAI2 itself. **(E) **Expression of SNAI2 mRNA in the same cohort, comparing tumors with high versus low ESR1/ERα levels. Asterisk (*) indicate statistically significant difference (*P *< 0.05) based on unpaired t-test with Welch correction of the log2-transformation of the signal intensities. **(F) **Analysis of the tumors with high ESR1/ERα expression for correlation between ESR1/ERα and SNAI2. The patient cohort for **D-F **was compiled from the publicly available GEO datasets GSE2034, GSE2603 and GSE12276 [37-39]. BCa, breast cancer; dox, doxycycline; E2, estradiol; ESR1/ER, estrogen receptor alpha; GEO, Gene Expression Omnibus; PCa, prostate cancer; RT-qPCR; reverse transcription - quantitative polymerase chain reaction; Runx2, runt related transcription factor 2; SNAI2, snail homolog 2.

Of the top 'common' genes, SNAI2 was of particular interest not only because its expression was the second most stimulated by Runx2 (6.6-fold) but also because it was strongly inhibited by E2 (-3.4-fold) (Table 2). Furthermore, the SNAI family of transcription factors is known to play a role in EMT [3-5]. By Western blot analysis, SNAI2 protein was detected in MCF7/Rx2^dox ^cells only after the induction of Runx2 (Figure 1A). Additional support for the regulation of SNAI2 by Runx2 was provided by RT-qPCR analysis of other cell types, namely C4-2B/Rx2^dox ^prostate cancer cells, in which SNAI2 was stimulated by dox-induced Runx2 (Figure 1B), and T47D/shRx2^dox ^BCa cells, in which SNAI2 expression was inhibited after dox-induced shRNA-mediated silencing of endogenous Runx2 (Figure 1C). We then sought evidence for the positive and negative regulation of SNAI2 by Runx2 and ERα, respectively, in BCa tumors. We compiled a cohort of 557 BCa patients from the publicly available datasets GSE2034, GSE2603 and GSE12276 [37-39] and initially investigated the correlation between SNAI2 expression and a Runx2 metagene, the latter defined as the average normalized expression of all genes that Runx2 stimulated by ≥ 2-fold in the MCF7/Rx2^dox ^system (except SNAI2 itself). As shown in Figure 1D, there was a positive correlation between expression of SNAI2 and the Runx2 metagene across the 557 BCa patient cohort. On the other hand, SNAI2 expression in the clinical specimens was negatively correlated with ERα/ESR1: tumors with low ESR1 expression (mostly histologically ER-negative) had significantly more SNAI2 transcripts than those with high ESR1 expression (Figure 1E), and the latter (mostly ER-positive tumors) exhibited a negative correlation between ESR1 and SNAI2 expression (Figure 1F). These data suggest that SNAI2 is stimulated by Runx2 and inhibited by E2 not only in the MCF7/Rx2^dox ^model (Table 2), but also in other cell lines, as well as in BCa tumors.

### E2 antagonizes Runx2-induced EMT and invasiveness *in vitro *

**Figure 2:**
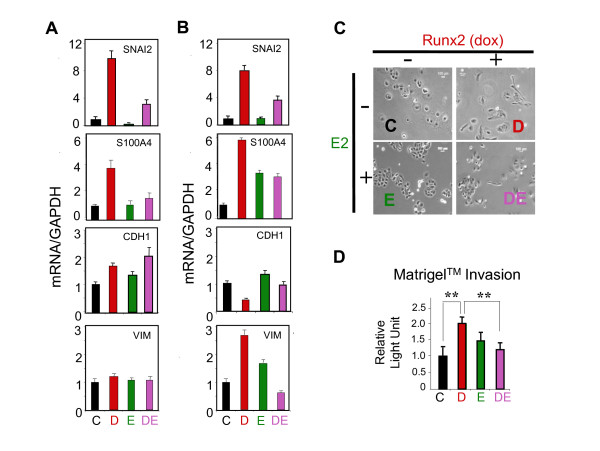
**E2 antagonizes Runx2-induced EMT markers and invasiveness of MCF7/Rx2^dox ^BCa cells**. (**A) **MCF7/Rx2^dox ^cells were maintained for two days in medium supplemented with CSS and then treated for two days with either vehicle control (C), dox (D), E2 (E), or both stimulants (DE), followed by RT-qPCR analysis of SNAI2, S100A4, vimentin (VIM) and E-cadherin (CDH1) mRNAs. (**B) **Relative expression of the same marker genes after treatment as in **A **for seven days. (**C) **Phase-contrast images of MCF7/Rx2^dox ^cells after seven days of treatments as indicated. (**D) **MCF7/Rx2^dox ^cells transduced with lentiviruses constitutively expressing firefly luciferase were placed in BD Biocoat™ Transwell inserts with Matrigel™ and treated for 24 hours as indicated. Luciferase activity in cells that invaded through the Matrigel™ membrane is presented relative to the average control value (Mean ± SD; *n *= 3; **P < 0.01). CSS, charcoal stripped serum; dox, doxycycline; E2, estradiol; EMT, epithelial-mesenchymal transition; RT-qPCR; reverse transcription - quantitative polymerase chain reaction; Runx2, runt related transcription factor 2; S100A4, S100 calcium binding protein A4; SNAI2, snail homolog 2.

Because SNAI2 has been implicated in EMT and in the invasive metastatic behavior of breast and other cancer cells [3,4] we employed the MCF7/Rx2^dox ^culture system to test the hypotheses that Runx2 induces and E2 antagonizes EMT and invasiveness, and that SNAI2 plays a role in these processes. We initially measured by RT-qPCR the expression levels of EMT and motility-related genes in MCF7/Rx2^dox ^cells treated with dox, E2, or both together. Consistent with the microarray results (Table 2), SNAI2 expression was increased by 9.7 fold and repressed by 4.6 fold in response to 48-hour treatment with dox and E2, respectively (Figure 2A). The mRNA for another well-established marker of motility and invasion, S100A4 [43,44], was also stimulated by Runx2. Although E2 did not affect basal S100A4 expression, it completely blocked the Runx2-mediated induction (Figure 2A). Despite the stimulation of SNAI2 and S100A4 by Runx2, these changes in gene expression were not accompanied by either noticeable morphological changes (data not shown) or a decrease in the epithelial marker E-cadherin (CDH1) or an increase in the mesenchymal marker vimentin (VIM) (Figure 2A). Taken together these results suggest that induction of Runx2 for two days at best initiated partial EMT in MCF7 cells.

We further assessed expression of EMT and motility-related genes seven days after Runx2 induction in MCF7/Rx2^dox ^cells. As shown in Figure 2B, this prolonged induction resulted in a more complete EMT. Not only were SNAI2 and S100A4 stimulated, CDH1 was downregulated and VIM was upregulated. Finally, microscopic examination of cultures seven days after Runx2 induction demonstrated a fibroblast-like and scattered morphology as compared to control cultures (Figure 2C). Thus, the partial EMT indicated by increased SNAI2 and S100A4 expression on day 2 (Figure 2A) developed into a more comprehensive EMT phenotype by day 7. More importantly, E2 significantly antagonized most of the Runx2-induced EMT characteristics. It blunted the Runx2-mediated effects on SNAI2 and CDH1 expression, and attenuated the Runx2-mediated effects on the expression of VIM and S100A4 (Figure 2B), as well as its effects on cell morphology (Figure 2C).

Finally, we measured the effects of Runx2 and E2 on BCa cell invasiveness. MCF7/Rx2^dox ^cells were transduced with lentiviruses constitutively expressing firefly luciferase and placed on top of Matrigel™ membranes within BD Biocoat™ inserts. Luciferase activity associated with cells that had crossed the membrane, a measure of invasiveness [30], was two-fold higher in cultures treated with dox to induce Runx2 as compared to controls, while co-treatment with E2 attenuated the Runx2-mediated invasiveness (Figure 2D). E2 alone had little, if any, effect on MCF7/Rx2^dox ^cell invasiveness through Matrigel™.

### SNAI2 plays a critical role in Runx2-mediated MCF7 cell invasion

**Figure 3:**
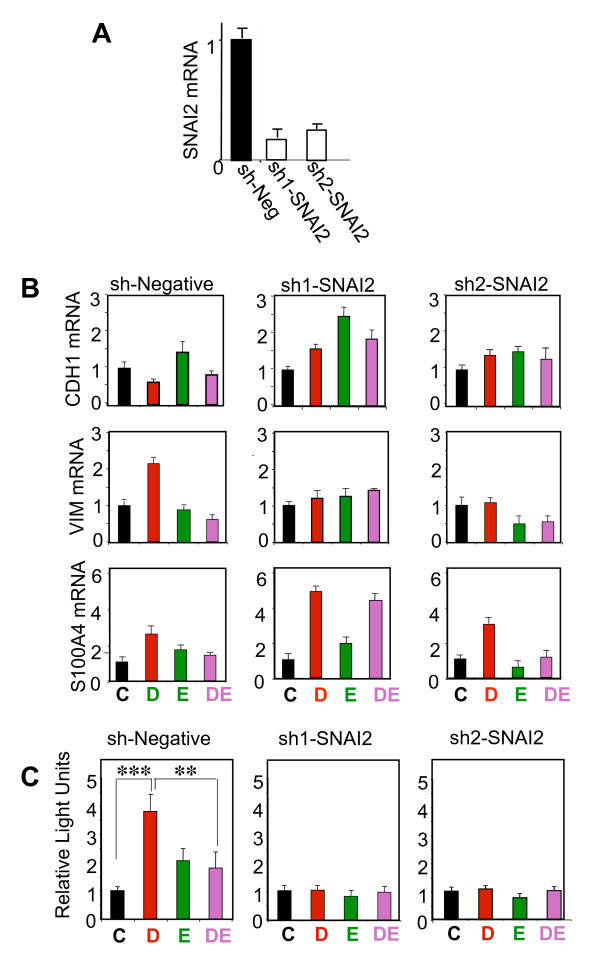
**SNAI2 is required for Runx2-mediated EMT**. (**A) **RT-qPCR analysis of SNAI2 mRNA in MCF7/Rx2^dox ^cells transduced with a control (sh-Neg) or each of two independent SNAI2 shRNA lentiviruses. (**B-C)**, MCF7/Rx2^dox ^cells transduced with sh1-SNAI2, sh2-SNAI2, or the negative control virus were treated with vehicle control (C), dox (D), E2 (E), or both stimulants (DE). The effects of SNAI2 knockdown on expression of the indicated EMT markers were assessed by RT-qPCR after seven days and the effects on invasion were assessed using Matrigel™-containing BD Biocoat™ Transwells as in Figure 2D, except dox treatment was initiated 6 hours prior to placing the cells in the transwell chambers. **, *P *< 0.01; ***, *P *< 0.005. Dox, doxycycline; EMT, epithelial-mesenchymal transition; E2, estradiol; RT-qPCR, reverse transcription - quantitative polymerase chain reaction; Runx2, runt related transcription factor 2; SNAI2, snail homolog.

We next tested the hypothesis that SNAI2 was required for Runx2-mediated EMT and invasion in MCF7/Rx2^dox ^BCa cells. To this end, MCF7/Rx2^dox ^BCa cells were transduced with lentiviruses expressing two independent shRNAs, sh1-SNAI2 and sh2-SNAI2, which target distinct regions of the SNAI2 transcript. Both shRNAs reduced SNAI2 mRNA levels by > 75% compared to cells expressing a control shRNA (Figure 3A). Cells were then treated with dox and/or E2, followed by RT-qPCR analysis of EMT markers and Matrigel™ invasion assays. Remarkably, SNAI2 knockdown by RNA interference abolished the Runx2-mediated decrease in CDH1 mRNA and increase in VIM mRNA, implicating SNAI2 in Runx2-mediated EMT (Figure 3B). Although SNAI2 silencing did not antagonize the stimulation of S100A4 by Runx2 (Figure 3B), it completely abrogated the ability of Runx2 to increase MCF7 cell invasion through Matrigel™ (Figure 3C).

### WNT and TGFβ signaling are required for Runx2-mediated stimulation of SNAI2 expression

**Figure 4:**
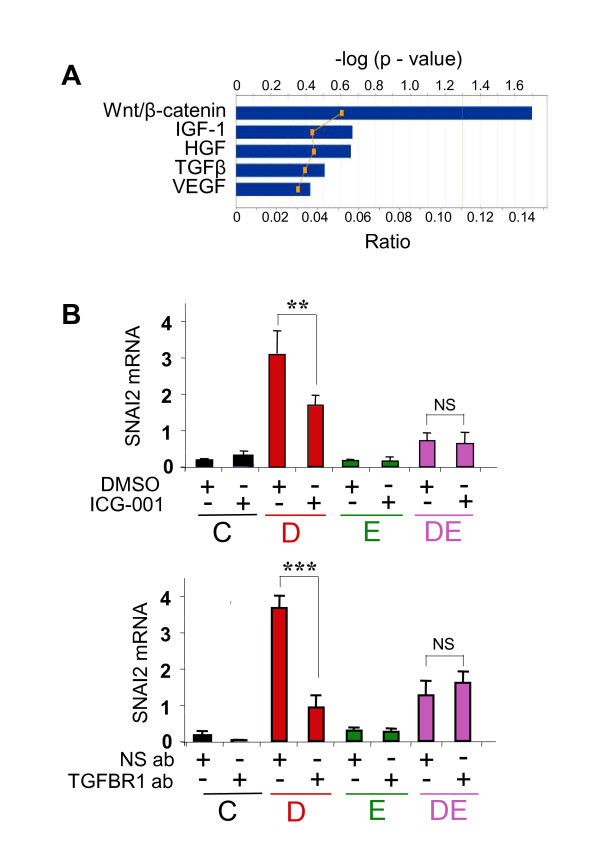
**WNT and TGFβ signaling are critical for Runx2-mediated stimulation of SNAI2 expression**. (**A) **The 530 'common' genes, which were regulated by both Runx2 and E2, were analyzed for enrichment for developmental and growth factor signaling pathways using the IPA™ software package. The bars and the upper scale indicate the log2-transformed significance values. The line graph and the bottom scale show magnitudes of enrichment represented as ratios. (**B) **MCF7/Rx2^dox ^cells were treated for 24 hours with vehicle control (C), dox (D), E2 (E) or both (DE), along with either 10 μM ICG-001, a small molecule inhibitor of Wnt signaling (top), or blocking antibody against the type I TGFβ receptor (bottom). DMSO and a non-specific antibody were used as respective controls. SNAI2 expression was measured by RT-qPCR and corrected for GAPDH. **, *P *< 0.01; ***, *P *< 0.005. DMSO, dimethyl sulfoxide; dox, doxycycline; E2, estradiol; GAPDH, glyceraldehyde-3-phosphate dehydrogenase; HGF, hepatocyte growth factor; IGF, insulin-like growth factor, IPA, Ingenuity Pathway Analysis; RT-qPCR; reverse transcription - quantitative polymerase chain reaction; Runx2, runt related transcription factor 2; TGF, transforming growth factor; SNAI2, snail homolog 2; VEGF, vascular endothelial growth factor.

EMT is regulated by various signal transduction pathways including WNT and TGFβ [45,46], and IPA™ analysis of the 530 'common' genes for representation of developmental and growth factor signaling pathways indicated enrichment of WNT/β-catenin pathway-related genes (Figure 4A). Both the WNT and TGFβ pathways have been also implicated in the regulation of SNAI2 expression [47,48]. Given that SNAI2 was required for Runx2-mediated EMT and invasiveness, we asked whether Runx2-mediated stimulation of SNAI2 expression involved WNT and TGFβ signaling pathways. To this end, MCF7/Rx2^dox ^cells were treated with dox, E2 or both, along with inhibitors of either WNT or TGFβ signaling. WNT signaling was inhibited by using ICG-001, a small molecule that interferes with the recruitment of CAAT-enhancer binding protein (CBP) to β-catenin/LEF/TCF complexes [35]. For TGFβ blockade, we used neutralizing antibodies against TGFBR1. As shown in Figure 4B, both ICG-001 and the anti-TGFBR1 antibody attenuated Runx2-induced SNAI2 expression. Interestingly, attenuation of the Runx2 response with either ICG-001 or the anti-TGFBR1 antibodies was no longer observed in cells co-treated with both dox and E2 (Figure 4B). Co-blockade of the WNT and TGFβ pathways was as effective as blocking each separately (data not shown). Thus, inhibition of either WNT or TGFβ signaling, or the presence of E2, suppressed Runx2-stimulated SNAI2 expression in MCF7 BCa cells. Neither WNT nor TGFβ interference affected Runx2-stimulated SNAI2 expression in the presence of E2. These results suggest that the WNT and TGFβ blockade may have targeted the same pathways by which E2 antagonizes Runx2 in BCa cells.

### Negative association of SNAI2 with metastasis-free survival in BCa patients

**Figure 5:**
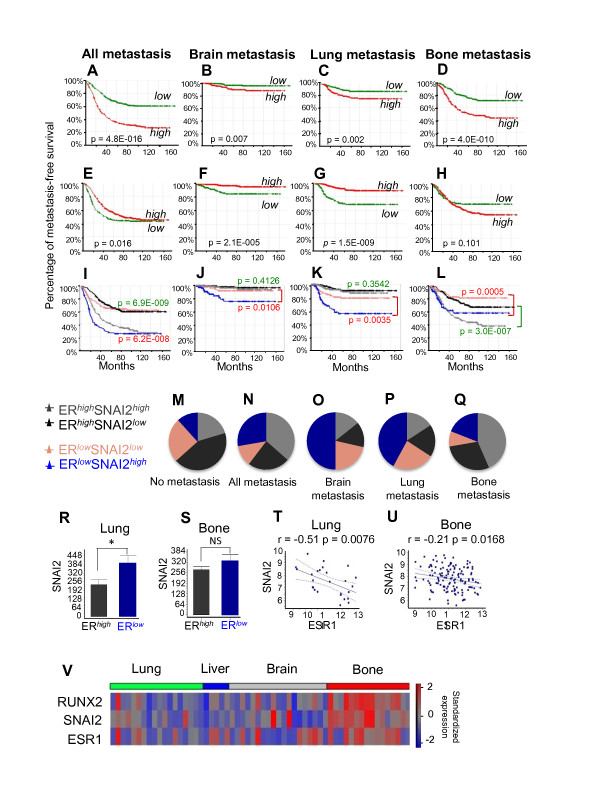
**Association of SNAI2 and ERα expression with metastasis**. A cohort of 557 BCa tumors compiled from the GEO datasets GSE2034, GSE2603 and GSE12276 [37-39], was dichotomized into groups expressing either high or low levels of either SNAI2 or ESR1/ERα, and Kaplan-Meier curves were constructed for the probability of overall or organ-specific metastasis-free survival in each group. (**A-D) **Kaplan-Meier curves of overall, brain, lung and bone metastasis-free survival in the cohort of 557 patients according to SNAI2 expression status. (**E-H) **Kaplan-Meier curves of overall, brain, lung and bone metastasis-free survival in the cohort according to ERα expression status.(**I-L) **Kaplan-Meier curves of overall, brain, lung and bone metastasis-free survival were generated for the 342 ERα-positive (black and grey curves) and 215 ERα-negative (blue and pink curves) tumors based on high versus low expression of SNAI2. Significance of association was determined by logrank test and the *P*-values are depicted for ERα-positive (green), and ERα-negative tumors (red). (**M-Q) **Expression status of SNAI2 and ERα in tumors that either have not relapsed **(M) **or metastasized to any tissue **(N)**, to brain **(O)**, to lung **(P)**, or to bone **(Q)**. **(R) **Expression of SNAI2 mRNA in lung-seeking tumors expressing ESR1/ERα at high versus low levels. The difference is significant (*, *P *< 0.05) based on unpaired t-test with Welch correction of the log2-transformed signal intensities. (**S) **Expression of SNAI2 mRNA in bone-seeking tumors expressing ESR1/ERα at high versus low levels. The difference is insignificant (NS, *P *= 0.23) based on unpaired t-test with Welch correction of the log2-transformed signal intensities. (**T-U) **Correlation between SNAI2 mRNA and ESR1/ERα mRNA in lung-seeking **(T) **and bone-seeking **(U) **tumors with high ERα. Values represent log2 transformed normalized signal intensities. (**V) **Standardized intensity plot of RUNX2, SNAI2 and ESR1/ERα gene expressions in 58 human BCa metastases. Expression data of metastatic tissues was extracted from the GEO dataset GSE14020 [9]. BCa, breast cancer; ESR1/ER, estrogen receptor alpha; GEO, Gene Expression Omnibus; RUNX2, runt related transcription factor 2; SNAI2, snail homolog 2.

We next determined the association between SNAI2 expression level and clinical BCa progression by constructing Kaplan-Meier curves for overall or site-specific metastasis-free survival of tumors expressing either high or low levels of SNAI2. Of the 557 patients compiled from the publicly available datasets GSE2034, GSE2603 and GSE12276 [37-39], 288 (52%) relapsed, with 184, 28 and 76 developing bone, brain and lung metastasis, respectively [38,39]. In cases of metastasis to multiple organs, each of the corresponding sites was included in the analysis. Overall, tumors expressing SNAI2 at high levels were more likely to relapse than those with low SNAI2 expression (Figure 5A-D). Accordingly, tumors which did not metastasize were mostly SNAI2^low ^(Figure 5M). The association between SNAI2 expression and metastasis was particularly strong for bone (*P *= 4.04E-010) (Figure 5D). These results suggest that high expression of SNAI2 in BCa primes tumors for metastasis in general and bone metastasis in particular.

### Anomalous high expression of SNAI2, Runx2 and ERα in bone-seeking BCa tumors

Suppression of SNAI2 expression in BCa cells by E2 (Figure 2A) may be related to the protective effect of ERα against metastasis. Indeed, consistent with previous reports [15,17-21], tumors with high ESR1 expression were less likely to metastasize in our cohort, particularly during the initial 20 to 40 months after surgery (Figure 5E). Analysis of the association between ESR1 expression and metastasis to specific sites, however, revealed an interesting exception. Whereas high ESR1 expression was negatively correlated with lung and brain metastasis (Figure 5F-G), it positively correlated with bone metastasis (Figure 5H). Thus, unleashing SNAI2 gene expression by loss of ERα is a plausible mechanism promoting metastasis in general; however, high expression of SNAI2 in cells that metastasize to bone occurs despite high ESR1 expression. Furthermore, SNAI2 expression was associated with lung and brain metastasis only for tumors expressing low levels of ESR1 (Figure 5J-K), whereas bone metastasis was associated with SNAI2 for tumors expressing ESR1 at either low or high levels (Figure 5L), suggesting weaker control of SNAI2 by ERα in bone-seeking tumors. Consequently, the combination of low ESR1 and high SNAI2 expression was predominant within lung and brain-seeking tumors (Figure 5O-P), whereas bone-seeking tumors were mostly those expressing both ESR1 and SNAI2 at high levels (Figure 5Q). Consistent with these observations, the negative correlation between ESR1 and SNAI2 expression was weaker in bone compared to lung-seeking tumors (Figure 5R-U; brain-seeking tumors were not analyzed because only eight of them were ER-positive). Finally, we investigated Runx2, SNAI2 and ESR1 gene expression in 58 BCa tumors derived from metastatic sites: 16 from bone, 19 from brain, 18 from lung and 5 from liver [9]. The standardized signal intensities plot showed preferential upregulation of Runx2, SNAI2 and ESR1 in the bone-derived biopsies (Figure 5V). The unusual concomitant high expression of ESR1 and Runx2/SNAI2 may reflect dominant regulation of Runx2/SNAI2 by E2-resistant signaling pathways that promote BCa bone metastasis.

## Discussion

Apart from promoting BCa progression, estrogen signaling has paradoxical anti-metastatic properties [15,17,19-21]. Potentially contributing to this, E2 antagonized the transcription factor Runx2, whose role in metastasis is being increasingly recognized [22,24,30-32]. Specifically, Runx2 promoted EMT and invasion of BCa cells *in vitro*, and this was antagonized by E2 (Figure 2). At the center stage of the opposing effects of Runx2 and E2 signaling on EMT and invasion was the transcription factor SNAI2. Consistent with previous reports [30,48,49], SNAI2 was stimulated by Runx2 and inhibited by E2 (Figure 1A and 2A); Runx2 no longer enhanced EMT and invasion after SNAI2 knockdown; SNAI2 expression in BCa biopsies positively correlated with a metagene that reports on Runx2 activity and negatively correlated with ERα mRNA (Figure 1B), and expression of SNAI2 was associated with BCa metastasis in general and bone metastasis in particular (Figure 5A-D).

SNAI2 likely plays different roles during various stages of BCa progression, and its ability to transcriptionally repress E-cadherin and induce EMT is well documented [3-5,49-51]. The role of SNAI2 downstream of Runx2 in BCa is reminiscent of the role of its homologue, SNAI1, in EMT and metastasis of breast, ovarian, colon, lung and squamous cell carcinomas [4]. However, SNAI1 is not stimulated by Runx2 in either MCF7 BCa [34] or C4-2B PCa cells [30]. Instead, it is SNAI2 that is strongly stimulated by Runx2 in BCa and PCa cells (Figure 2). Furthermore, SNAI2 and not SNAI1 was repressed by E2 in our study (data not shown), and SNAI2 and not SNAI1 exhibited an inverse correlation with ERα and E-cadherin in human BCa tumors [49]. Like Runx2, Twist1 has recently been shown to induce SNAI2 expression, and SNAI2 was essential for Twist1-mediated EMT of human mammary epithelial cells [50]. Thus, depending on context, either SNAI1 or SNAI2 regulate BCa metastasis, with the latter mediating the pro-metastatic activity of Runx2 and Twist1 as well as the anti-metastatic activity of E2.

It remains to be investigated whether Runx2 directly regulates SNAI2 transcription. On the one hand, there are several Runx-binding motifs upstream of the SNAI2 transcription start site (Additional file 3: Supplemental Figure 1A). However, Runx2 ChIP-seq analysis in C4-2B cells, where SNAI2 expression is also stimulated by Runx2 (Figure 1B), did not suggest occupancy of Runx2 at these SNAI2 upstream Runx motifs [52]. Instead, we believe that Runx2 indirectly stimulates SNAI2 expression via modulation of ETS signaling. This hypothesis is based on the observation that Runx2 down-regulated expression of SPDEF (Additional file 3: Supplemental Figure 1B and [30]), a transcriptional inhibitor belonging to the ETS family, which suppresses tumor progression and metastasis [53] in part through direct inhibition of SNAI2 expression [54]. Indeed, our Runx2 ChIP-seq analysis in C4-2B/Rx2^dox ^cells [52] demonstrated strong occupancy of the SPDEF transcription start site by Runx2 (Additional file 3: Supplemental Figure 1C). We are currently investigating how such Runx2 occupancy represses SPDEF transcription, and which of the many ETS sites upstream of the SNAI2 transcription start site (Additional file 3: Supplemental Figure 1A) mediate its repression by SPDEF and/or its activation by other members of the ETS family. Regardless of the precise mechanism by which Runx2 controls SNAI2, expression of the two is tightly correlated in BCa tumors (Figure 1D). In fact, much of the reported association between Runx2 expression and BCa metastasis [22] is attributable to SNAI2. Indeed, the results of our independent analysis validating the association of Runx2 with metastasis (Additional file 4) are remarkably similar to those demonstrating the association of SNAI2 with metastasis (Figure 5).

The anti-Runx2/SNAI2, anti-metastatic properties of E2 signaling in BCa cells may be relevant for early *in vivo *metastatic events (Figure 5E), and possibly to targeting BCa metastasis to non-osseous tissues (Figure 5F-G). However, they appear less relevant for bone metastasis. Whereas SNAI2 expression is associated with bone-seeking primary BCa tumors and BCa bone metastasis even more strongly than with lung or brain metastasis, ERα is negatively associated with only non-osseous metastasis (Figure 5). In fact, consistent with previous reports [9,55], the correlation between ERα expression and bone metastasis was positive, not negative in our cohort (Figure 5H); BCa tumors that metastasized to bone were mostly ER-positive (Figure 5Q) and BCa bone metastases co-expressed high levels of Runx2/SNAI2 and ERα (Figure 5V). The bone-seeking property that ERα bestows on BCa cells may be counteracted during the first two years after surgery by the general anti-metastatic property of estrogens, resulting in a total neutral effect, but at the end it is the former that prevails (Figure 5H). Possibly, activation of bone-seeking pathways in primary BCa cells, such as Src1 [8,9], results in the stimulation of SNAI2 and other Runx2-regulated genes despite high levels of ERα.

## Conclusions

Runx2 stimulates EMT and invasiveness in MCF7 BCa cell cultures, adding to the mounting evidence for its role in metastasis. Our data suggest that E2 attenuates BCa metastasis by antagonizing Runx2. Indeed, EMT and invasiveness of MCF7 cells were severely compromised by E2 specifically after induction of Runx2. The linkage between the pro- and anti-metastatic properties of Runx2 and E2, respectively, is attributable in part to SNAI2, which is upregulated by Runx2 and downregulated by E2. Furthermore, we observe a strong positive association between Runx2 and SNAI2 expression, a negative association between ESR1 and SNAI2, and a positive correlation between SNAI2 expression and metastasis in BCa tumors. These observations are consistent with the hypothesis that unopposed stimulation of Runx2 target genes such as SNAI2 in ER-negative tumors contributes to their aggressive metastatic phenotype. However, the relationship between ER expression and metastasis is more complicated in bone-seeking tumors. Here, the negative correlation between SNAI2 and ER is weak, and the anti-metastatic property of ER is likely masked by an opposite property, which ultimately prevails. Mimicking ER signaling to specifically antagonize Runx2 and SNAI2 offers a research avenue towards the development of novel therapeutic approaches for the management of BCa patients who fail first line therapy.

## Abbreviations

BCa: breast cancer; DAVID: Database for Annotation: Visualization and Integrated Discovery; dox: doxycycline; E2: estradiol; EMT: epithelial-mesenchymal transition; ERα: estrogen receptor alpha; IPA: Ingenuity Pathway Analysis; PCa: prostate cancer; RMA: Robust Multiarray Averaging; SNAI2: snail homolog 2; SPDEF: SAM pointed domain-containing ets transcription factor.

## Competing interests

The authors declare that they have no competing interests.

## Authors' contributions

NOC, SKB and GHL performed the experiments. NOC and YBC performed the bioinformatics analyses. NOC, MK, ZB, DT and BF designed the study and wrote the manuscript. All authors read and approved the final manuscript.

## Supplementary Material

Additional file 1 **Oligonucleotide sequences used in this study**. List of primer pair sequences for qRT-PCR and shRNA sequences. Click here for file

Additional file 2 **The complete list of the 'common' genes**. The complete list of the 'common' genes, independently regulated by Runx2 and E2 in MCF7/Rx2^dox ^cells are depicted along with respective fold changes and p values. Click here for file

Additional file 3 **Potential Mechanisms Linking Runx2 to SNAI2**. Based on the observation that Runx2 down-regulated expression of SPDEF and strong occupancy of the SPDEF transcription start site by Runx2 we hypothesize that Runx2 indirectly stimulates SNAI2 expression in part via modulation of ETS signaling. Click here for file

Additional file 4 **Association of Runx2 activity with metastasis**. Runx2 activity was associated with both osseous and non-osseous metastases. Bone-seeking BCa tumors exhibit an unusual combination of high RUNX2 activity and high ERα levels. Click here for file
