# Supplementary material for: Regulation of breast cancer metastasis by Runx2 and estrogen signaling: the role of SNAI2
Source: Breast Cancer Res. 2011 Dec 9;13(6):R127. doi: 10.1186/bcr3073 (PMC3326569; doi:10.1186/bcr3073)
Supplement: Additional file 3 — Potential Mechanisms Linking Runx2 to SNAI2. Based on the observation that Runx2 down-regulated expression of SPDEF and strong occupancy of the SPDEF transcription start site by Runx2 we hypothesize that Runx2 indirectly stimulates SNAI2 expression in part via modulation of ETS signaling. [file bcr3073-S3.PPT]

## Slide 1
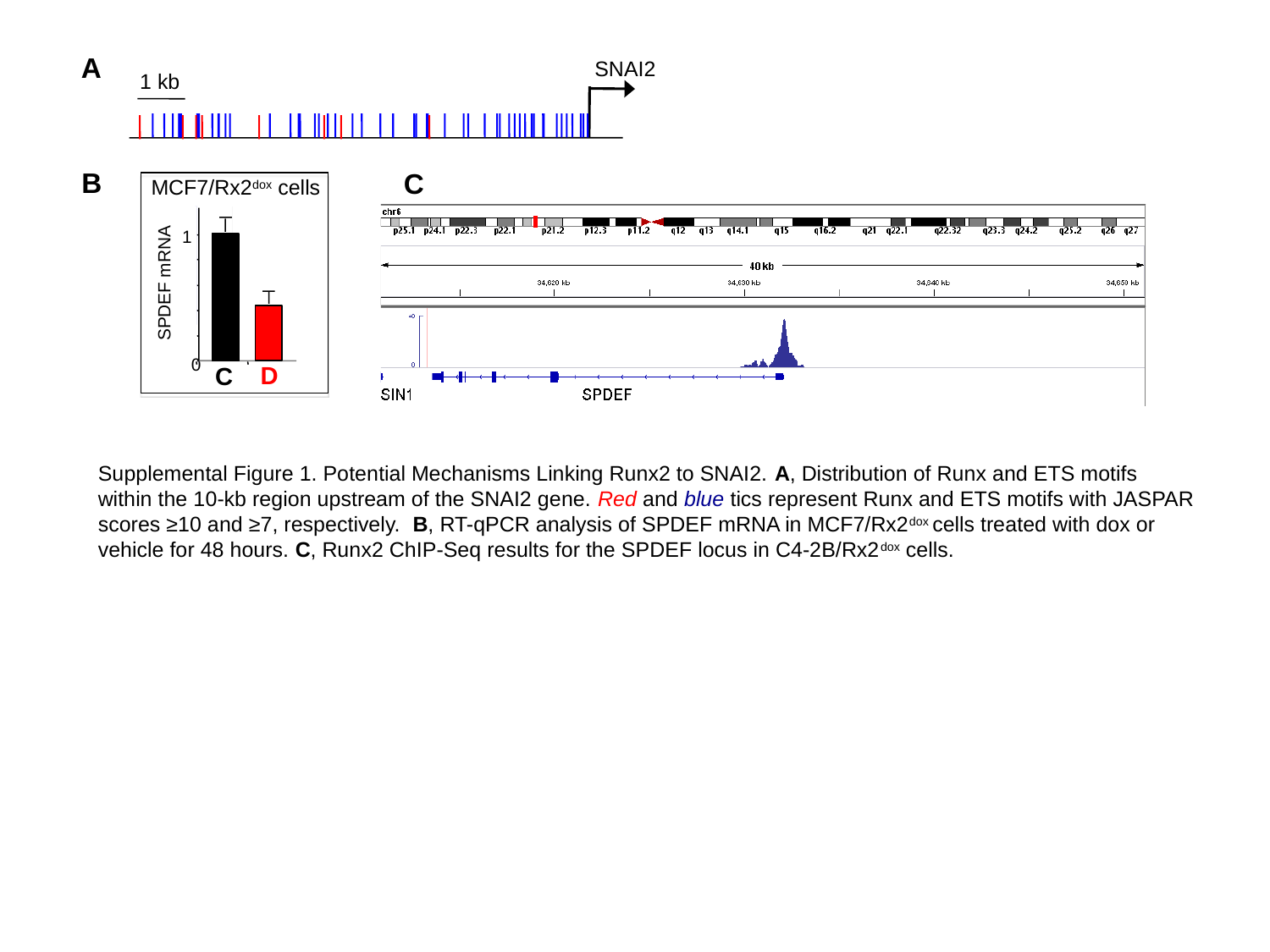

A
SNAI2
1 kb
B
C
MCF7/Rx2dox cells
0
1
SPDEF mRNA
D
C
Supplemental Figure 1. Potential Mechanisms Linking Runx2 to SNAI2. A, Distribution of Runx and ETS motifs within the 10-kb region upstream of the SNAI2 gene. Red and blue tics represent Runx and ETS motifs with JASPAR scores ≥10 and ≥7, respectively. B, RT-qPCR analysis of SPDEF mRNA in MCF7/Rx2dox cells treated with dox or vehicle for 48 hours. C, Runx2 ChIP-Seq results for the SPDEF locus in C4-2B/Rx2dox cells.
