# Supplementary material for: Regulation of breast cancer metastasis by Runx2 and estrogen signaling: the role of SNAI2
Source: Breast Cancer Res. 2011 Dec 9;13(6):R127. doi: 10.1186/bcr3073 (PMC3326569; doi:10.1186/bcr3073)
Supplement: Additional file 4 — Association of Runx2 activity with metastasis. Runx2 activity was associated with both osseous and non-osseous metastases. Bone-seeking BCa tumors exhibit an unusual combination of high RUNX2 activity and high ERα levels. [file bcr3073-S4.PPTX]

## Slide 1
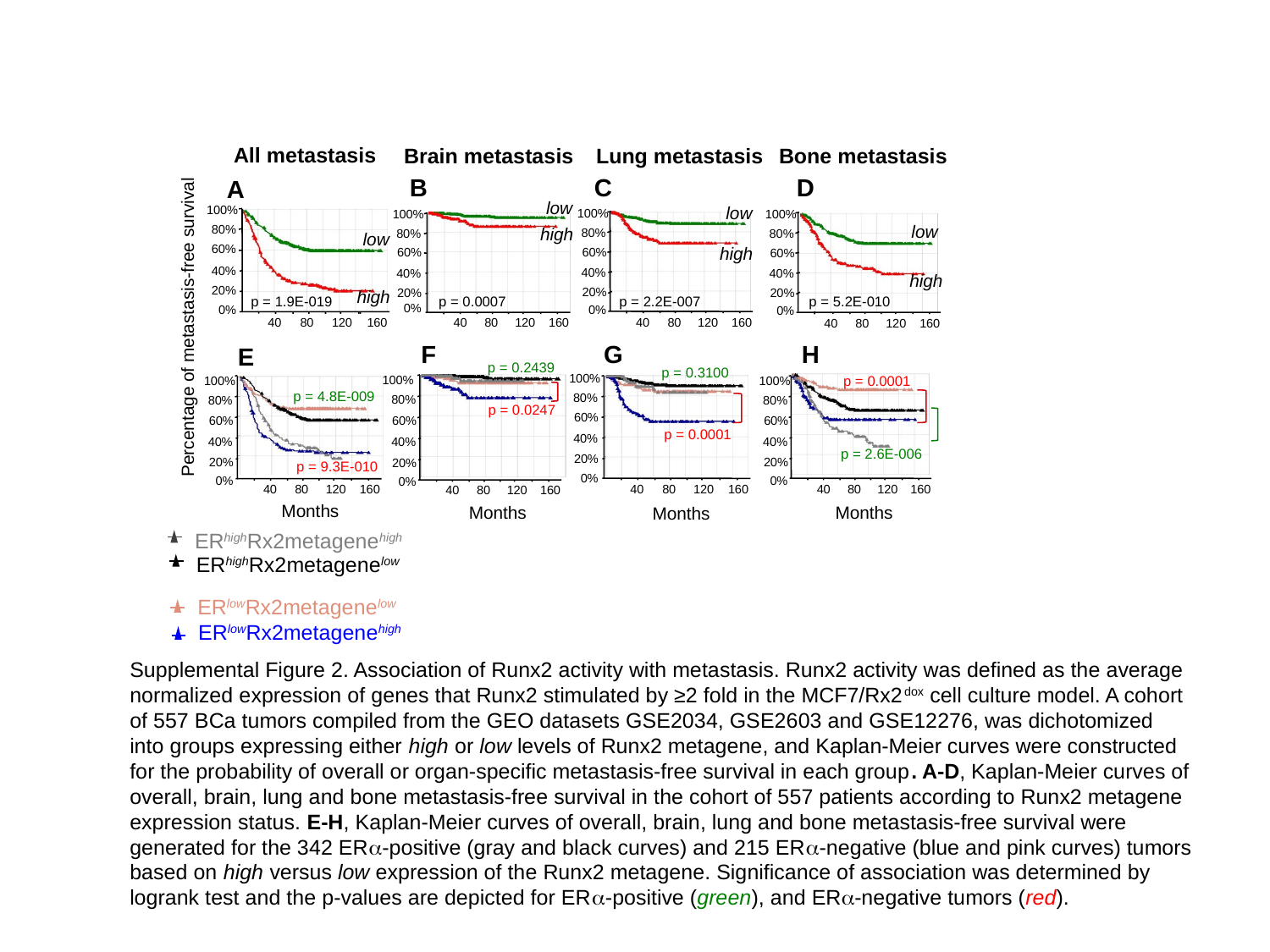

All metastasis
Brain metastasis
Bone metastasis
Lung metastasis
B
C
D
A
low
low
100%
80%
60%
40%
20%
0%
100%
80%
60%
40%
20%
0%
40
80
120
160
high
p = 2.2E-007
100%
80%
60%
40%
20%
0%
40
80
120
160
100%
80%
60%
40%
20%
0%
40
80
120
160
low
high
low
high
high
p = 0.0007
p = 1.9E-019
p = 5.2E-010
Percentage of metastasis-free survival
40
80
120
160
F
G
H
E
p = 0.2439
p = 0.3100
100%
80%
60%
40%
20%
0%
40
80
120
160
100%
80%
60%
40%
20%
0%
40
80
120
160
p = 0.0001
100%
80%
60%
40%
20%
0%
40
80
120
160
100%
80%
60%
40%
20%
0%
40
80
120
160
p = 4.8E-009
p = 0.0247
p = 0.0001
p = 2.6E-006
p = 9.3E-010
Months
Months
Months
Months
ERhighRx2metagenehigh
ERhighRx2metagenelow
ERlowRx2metagenelow
ERlowRx2metagenehigh
Supplemental Figure 2. Association of Runx2 activity with metastasis. Runx2 activity was defined as the average normalized expression of genes that Runx2 stimulated by ≥2 fold in the MCF7/Rx2dox cell culture model. A cohort of 557 BCa tumors compiled from the GEO datasets GSE2034, GSE2603 and GSE12276, was dichotomized into groups expressing either high or low levels of Runx2 metagene, and Kaplan-Meier curves were constructed for the probability of overall or organ-specific metastasis-free survival in each group. A-D, Kaplan-Meier curves of overall, brain, lung and bone metastasis-free survival in the cohort of 557 patients according to Runx2 metagene expression status. E-H, Kaplan-Meier curves of overall, brain, lung and bone metastasis-free survival were generated for the 342 ERα-positive (gray and black curves) and 215 ERα-negative (blue and pink curves) tumors based on high versus low expression of the Runx2 metagene. Significance of association was determined by logrank test and the p-values are depicted for ERα-positive (green), and ERα-negative tumors (red).
